# Adenotonsillectomy Versus Watchful Waiting for Children with Obstructive Sleep Apnea Syndrome: A Systematic Review with Meta-Analysis

**DOI:** 10.1007/s12070-024-04738-0

**Published:** 2024-05-20

**Authors:** Alexios Tsikopoulos, Konstantinos Tsikopoulos, Fotios Dilmperis, Sofia Anastasiadou, Konstantinos Garefis, Athanasios Fountarlis, Stefanos Triaridis

**Affiliations:** 1 1st Department of Otorhinolaryngology-Head and Neck Surgery, AHEPA University General Hospital, Aristotle University of Thessaloniki, Kiriakidi 1, Thessaloniki, 546 21 Greece; 2 Department of Orthopedics, 424 Army General Training Hospital, Thessaloniki, Greece; 3 School of Medicine, Aristoteles University of Thessaloniki, Thessaloniki, Greece; 4 2nd Department of Otorhinolaryngology-Head and Neck Surgery, Papageorgiou University General Hospital, Aristotle University of Thessaloniki, Thessaloniki, Greece; 5 Department of Otorhinolaryngology, University General Hospital of Larissa, University of Thessaly, Larissa, Greece

**Keywords:** Systematic review, Meta-analysis, Adenotonsillectomy, Obstructive sleep apnea

## Abstract

The treatment of children with Obstructive Sleep Apnea Syndrome (OSAS) remains a point of debate among otorhinolaryngologists worldwide. This study aims to comparatively assess the clinical outcomes of adenotonsillectomy (ATE) and watchful waiting in children with OSAS. We searched the databases of PubMed, Scopus, and Cochrane Central Register of Controlled Trials (CENTRAL), until the 1st of October 2023. Comparative trials assessing the efficacy of adenotonsillectomy and watchful waiting in children with OSAS were considered. The primary outcome measure was the apnea-hypopnea index (AHI), and secondary outcomes included quality of life as measured by OSA-18 score, and mean SpO2 levels. A subgroup analysis evaluating the changes in AHI depending on the severity of the initial disease was also executed. We conducted a random-effects pairwise meta-analysis of change scores to include randomized and non-randomized controlled trials. The quality assessment was carried out by using the Cochrane risk of bias tool for randomized and the ROBINS-I tool for non-randomized trials, respectively. Two randomized and five non-randomized trials were included. There was a statistically significant difference regarding AHI in favor of the ATE group compared to the watchful waiting group (Standardized mean difference [SMD] was − 0.60, 95%CI −0.79 to −0.41, *p* < 0.001). Likewise, a statistically significant decrease in change scores for OSA-18 between ATE and watchful waiting was noted (SMD was − 0.79, 95%CI −0.97 to −0.61, *p* < 0.001). On the other hand, there was no significant difference when ATE and watchful waiting groups were compared for SpO2 levels between each other (SMD was 0.52, 95%CI −1.53 to 2.56, *p* < 0.62). In the subgroup analysis assessing mild OSAS, there was a significant difference in favor of ATE compared to watchful waiting (SMD was −0.91, 95%CI −1.35 to −0.47, *p* < 0.0001). For mild to moderate OSAS, similar results were noted favoring ATE as well (SMD was − 0.53, 95%CI −0.87 to −0.19, *p* < 0.003). This study provides evidence of moderate strength supporting the superiority of ATE over the watchful waiting approach in terms of AHI and OSA-18. This also appears to be the case for AHI in children with mild and mild to moderate OSAS.

**Supplementary Information:**

The online version contains supplementary material available at 10.1007/s12070-024-04738-0.

## Introduction

Obstructive Sleep-Disordered Breathing (OSDB) is an umbrella term encompassing medical conditions, in which either partial or complete cessation of breathing occurs several times throughout the night, leading to poor concentration, daytime sleepiness, and chronic fatigue that negatively affects daily activities and quality of life [1, 2]. From an epidemiological point of view, OSDB occurs in 1 to 5% of children and its severity varies from simple snoring to Obstructive Sleep Apnea Syndrome (OSAS) [3, 4]. Although it can occur at any age, its prevalence is higher in children between two and six years of age. On the other hand, no significant differences in prevalence between males and females have been documented [3, 5, 6].

Pediatric OSAS is characterized by intermittent partial or complete collapse of the upper airway during sleep, resulting in reduction (hypopnea) or complete cessation (apnea) of airflow leading to arousal and hypoxia [7]. Although apnea is generally defined as the complete cessation of oronasal airflow for at least 10 s, in the pediatric population, any respiratory pause is considered apnea, regardless of the duration [8].

More specifically, OSAS is a multifactorial condition that might be linked to craniofacial anomalies, obesity, hypothyroidism, neuromuscular disorders, asthma, allergic rhinitis, and Down syndrome [9, 10]. In children, adenotonsillar hypertrophy is the most common cause of OSAS [11]. There is a significant correlation between tonsillar volume and OSBD severity [12].

Without timely diagnosis and intervention, pediatric OSAS can lead to serious clinical sequelae, including behavioral abnormalities, neurocognitive impairment, learning disabilities, cardiovascular and pulmonary hypertension, endocrine metabolic disorders, maxillofacial dysplasia (adenoid faces), growth development restriction, and an increase in the risk of cardiovascular events in adulthood [10, 13].

Clinical evaluation of pediatric OSAS consists of careful history taking, clinical examination, and eventually endoscopic evaluation. History taking and clinical examination have been reported to have positive predictive value for diagnosis of OSAS of 65% and 46%, respectively [14]. However, although history taking and physical examination are crucial in establishing OSDB diagnosis, the latter is normally confirmed by PSG [15]. Presently, PSG represents the gold standard for the diagnosis of pediatric OSAS [8]. According to the American Academy of Otolaryngology-Head and Neck Surgery Foundation (AAO-HNSF) guidelines, PSG should be performed prior to tonsillectomy in children affected by OSBD aged 2 to 18 years [16].

Pediatric OSAS sometimes, but not always, results in dips in hemoglobin saturation [17]. Hence, for the initial diagnosis of OSBD and OSAS, nocturnal pulse oximetry can also serve as a simple diagnostic tool, given its high positive predictive value (97%), its easy accessibility and applicability, as well as its relatively low cost. Furthermore, this method could be potentially useful for the selection of the most PSG-deserving children, even if this measurement is specific and not sensitive enough [17, 18].

In sleep study monitoring, the frequency of apneas and hypopneas per hour of sleep (apnea–hypopnea index [AHI]) is the key measure to define and stratify the severity of OSAS [19]. In children, the detection of a single apnea or hypopnea episode per hour is considered pathological (AHI > 1). Three degrees of OSAS severity have been identified in relation to the AHI in the pediatric population: mild AHI 1–4, moderate AHI 5–9, and severe AHI ≥ 10 [8].

Adenotonsillectomy (ATE) is currently one of the first-line treatments for pediatric OSAS. Its clinical effect is particularly significant in children with moderate or severe OSAS [20]. However limited evidence currently exists about the benefit of ATE for children with mild OSAS [21].

The aim of the present systematic review and meta-analysis is to compare the clinical efficacy of ATE with a watchful waiting strategy in the pediatric population with OSAS. In addition, we sought to investigate separately the cases of mild or mild to moderate OSAS in children, as this remains the main area of controversy.

## Methods

The present systematic review with meta-analysis was registered with PROSPERO (CRD42022320656). We also used the Preferred Reporting Items for Systematic Reviews and Meta-Analyses (PRISMA) [22].

### Eligibility Criteria

Both randomized and non-randomized trials comparing watchful waiting with ATE exclusively in pediatric patients with OSAS were included in the systematic review. Of note, all three degrees of OSAS were considered in the analysis.

### Literature Search

Two investigators (A.T. and S.A.) performed a thorough literature search in a blinded manner to identify published and unpublished studies comparing adenotonsillectomy with watchful waiting in children with OSAS. The databases of PubMed, Scopus, and Cochrane Central Register of Controlled Trials (CENTRAL) were assessed, until the 1st of October 2023 for trials comparatively assessing the efficacy of adenotonsillectomy and watchful waiting in children with OSAS. Of note, no language restrictions were applied. Moreover, we also searched the registries of ClinicalTrials.gov, International Standard Randomized Controlled Trial Number (ISRCTN), and Australian New Zealand Clinical Trials Registry (ANZCTR) for completed unpublished trials up to the same date. Furthermore, reference lists from relevant articles were manually searched to identify additional studies. The final search was performed using Ovid Search Tool (Ovid R by Wolters Kluwer) with the cooperation of a licensed librarian. The search strategy is presented in Supplemental File 1.

### Selection of Studies

Two independent review authors (A.T. and S.A.) screened the titles and abstracts of articles retrieved through the systematic literature search to identify potentially relevant records independently. Following de-duplication, the titles and abstracts of the remaining papers were screened for eligibility. The full text of the remainder of the articles was assessed against our inclusion criteria. Any discrepancies between the two investigators were discussed and resolved through consensus.

### Extraction of Data

Data extraction was independently conducted by two reviewers (A.T. and S.A.). From each eligible study, we collected the year of publication, country, study design, follow-up period, and intervention groups. We also extracted data about characteristics such as age, sex, inclusion and exclusion criteria, treatment protocols, and outcomes measured.

### Outcome Assessment

The primary endpoint of this systematic review was the AHI since it is the main parameter of PSG used to determine OSAS and is considered the baseline indicator of OSAS. OSA-18 score and mean SpO2 levels were considered secondary outcomes. Further outcomes were not able to be synthesized in the present study because of a lack of available data.

### Quality Assessment

Two researchers (A.T. and S.A.) conducted the quality appraisal of the randomized and non-randomized trials included in the analysis, using the Cochrane Collaboration’s ‘risk of bias’ tool [23] and ROBINS-I tool [24], respectively (Tables 1 and 2).

**Table 1:** Studies characteristics. Author names, year of publication, study design, intervention groups, age and sex of the participants, follow-up period, number of completers and losses of patients in the follow-up are presented for the included studies

Study (year)	Country	Study design	Intervention groups [patients] (AT: observation)	Age [range] (years)	Mean age at first PSG (AT: observation)	Males/females (AT: observation)	Follow-up period	Number of patients (completers)	Losses in follow-up
Huang (2007)	Taiwan	Non-randomized open-labeled trial	*n* = 25:*n* = 14	6 to 12	8.08 ± 1.28:8.07 ± 2.30	23/2:12/2	6 months	39	3
Ben-Israel (2010)	Israel	Cohort study	*n* = 14:*n* = 6	Pre-puberty age range	6.4 ± 2.5:5.4 ± 2.2	7/7:4/2	Time between the 1st and 2nd PSG studies: 10.5 ± 7 (range 4–25) and 19 ± 20 (range 6–62) months for the AT and observation group, respectively. For the AT group, post-PSG studies were repeated 5.4 ± 4 (range 1.5–18) months after the surgery.	20	0
Volsky (2013)	USA	Prospective, nonrandomized trial	*n* = 30:*n* = 34	3 to 16	80.8 ± 33.7:80.0 ± 41.9 (months)	16/14:20/14	8 months	64	49
Marcus (2013)	Canada	Parallel-group RCT	*n* = 194:*n* = 203	5 to 9	6.5 ± 1.4:6.5 ± 1.4	106/97:89/105	7 months	397	64
Trosman (2016)	USA	Retrospective Chart Review	*n* = 18:*n* = 44	9 months to 9 years	3.5:3.1	11:7/ 26:18	Average time from surgery to follow-up PSG for patients undergoing adenotonsillectomy 10.3 months.	62	0
Fehrm (2020)	Sweden	RCT	*n* = 29:*n* = 31	2 to 4	39 ± 8:37 ± 11 (months)	ATE: 15:14/19:12	6 months	60	7
Martinez-Ruiz de Apodaca (2020)	Spain	Cohort study	*n* = 201:*n* = 41	14 to 157 months	49.97 ± 21.3:51.3 ± 16.72 (months)	40% female	12 ± 3 months	242	75

PSG = Polysomnogrpahy, RCT = Randomized Controlled Trial

**Table 2:** Studies characteristics. Author names, year of publication, treatment protocols, outcomes, inclusion and exclusion criteria of the studies are presented

Study (year)	Treatment protocol	Outcomes	Inclusion criteria	Exclusion Criteria
Huang (2007)	Comparison of ADHD children with mild OSA subjected to AT performed by the same otolaryngologist, with children with OSA without AT	ΑΗΙ (event/hour), AI (event/hour), SI (event/hour), ODI (event/hour), Mean SO2, Arousal counts, REM	(1) Diagnosed ADHD according to DSM-IV criteria (2) AHI > 1 (3) Age 6–12 years (4) Wechsler Intelligence Scale for Children IQ testing Score of 70 or more (5) Confirmed adenotonsillar hypertrophy by pediatric otolaryngologist	(1) PSG diagnosis of PLMI > 5 (2) History of seizure disorder (3) Systemic disease (4) Other physical disease (5) Major psychiatry disease
Ben-Israel (2010)	Consecutive recruitment and inclusion of children with PSG-proven OSA for the AT group. Retrospective recruitment of children with PSG-proven OSA with no previous surgery or CPAP treatment for the observation group.	AHI (events/hour), SpO2, REM, SWA energy and slow wave slope	(1) Healthy children referred for possible OSA (2) For observation group, children with PSG-proven OSA with no previous surgery or CPAP treatment for the observation group.	(1) Chronic medical illnesses (2) Genetic disorders (3) Facial anomalies (4) Poor quality EEG signals
Volsky (2013)	Caregivers chose between management options and assigned patients to AT group or to observation group. No differences between the 2 groups in terms of demographic and polysomnographic data. No additional medical management with intranasal steroids and montelukast.	AHI, O2 nadir, Arousal index, Peak end-tidal CO2, OSA-18, CHQ PF-28	(1) Age 3 to 16 years (2) New diagnosis of mild OSA, defined by AHI from 1 to 5 on full-night PSG (3) Tonsillar hypertrophy on physical examination (Grade 2, 3, and 4 tonsils in the Brodsky grading scale)	(1) Craniofacial abnormalities (2) Cerebral palsy (3) Trisomy 21 (4) Prior adenotonsillar surgery (5) Children with parents not speaking English
Marcus (2013)	Random assignment to AT (surgery within 4 weeks after randomization) or to a strategy of watchful waiting.	(1) OSA-18 assessment tool (2) PSQ-SRBD score (3) Modified Epsworth Sleepiness Scale scores (4) PedsQL scores (5) ODI (6) Mean ODI score (7) Mean percentage of sleep time with CO2 (8) Conners Rating Scale scores, Developmental Neuropsychological Assessment (NEPSY), Behavior Rating Inventory of Executive Function [BRIEF] Global Executive Composite T score, PSQ-SRBD (9) Epworth Sleepiness Scale modified for children (10) OSA-18 assessment tool, 11) AHI	(1) Age 5 to 9 years (2) OSA without prolonged oxyhemoglobin desaturation, (3) Suitable candidates for adenotonsillectomy	(1) Recurrent tonsillitis (2) a z score based on the body-mass index (the weight in kilograms divided by the square of the height in meters) of 3 or more, and (3) medication for ADHD
Trosman (2016)	Initial PSG and assignment of the patients to observation group or to tonsillectomy and adenoidectomy group, based on individualized clinician’s practice and parental input on impact on the child.	AHI events	(1) Children under the age of 10 years at the time of the initial PSG (2) 2 or more PSG’s performed (3) mild OSA, defined as AHI, subsequently referred to as AHI, between 1 and 5	(1) Age 10 years or older at the time of initial PSG (2) Previous upper aerodigestive tract surgery, including adenoidectomy alone or combined adenotonsillectomy (3) Use of positive pressure ventilation, including CPAP, and dental appliances
Fehrm (2020)	PSG in all referred children with a history of habitual snoring, apneas, and/or restless sleep. Random assignment of children to either ATE or watchful waiting. Surgery within approximately 3 months of the baseline PSG. Removal of the tonsils by blunt extracapsular dissection, and removal of the adenoid with a ring knife or coblation.	(1) AHI score (2) Central AHI (3) Rapid eye movement AHI (4) ODI (using the ≥ 3% desaturation criterion) (5) Respiratory distress index (6) Mean oxygen saturation (7) Lowest oxygen saturation level (8) Sleep stages (9) Total sleep time (10) Wake after sleep onset 11) Sleep efficiency 12) OSA–18 questionnaire 13) Sleep disturbance score 14) VAS QoL	(1) Age 2 years or older and younger than 5 years (2) History or symptoms of habitual snoring, apneas, and/or restless sleep (3) Mild to moderate OSA, defined as an AHI score of 2 or more and less than 10 (mild OSA, OAHI ≥ 2 and < 5; moderate OSA, AHI ≥ 5 and < 10) (4) Tonsilar hypertrophy of 2 to 4 according to Brodsky (5) Parents with sufficient knowledge of Swedish to understand the written information and answer the questionnaires	(1) Craniofacial abnormality (2) Neuromuscular disease (3) Chromosomal abnormality (4) Previous adenotonsillar surgery (5) Bleeding disorder (6) Cardiopulmonary disease (e.g., heart valve disease, cystic fibrosis, and asthma; mild infection-related asthma was not excluded)
Martinez-Ruiz de Apodaca (2020)	Comparison of children with OSA subjected to adenoidectomy and pharyngeal surgery (tonsillectomy or tonsilotomy and adenopharyngoplasty) with patients whose parents refused any treatment and were subjected to surveillance without intervention.	(1) AHI (2) Mean oxygen saturation (3) Time spent with arterial oxygen saturation < 90% (4) PSQ-SRBD (5) Clinical questionnaire described by Esteller et al.	(1) Age 1 to 13 years (2) AHI ≥ 3/h (3) No previous treatment	N/A

ADHD = Attention Deficit Hyperactivity Disorder, ATE = Adenotonsillectomy, AI = Apnea Index, AHI = Apnea Hypopnea Index, CPAP = Continuous Positive Airway Pressure, CHQ PF-28 = Child Health Questionnaire Parent Form 28, DI = Desaturation Index, EEG = Electroencephalography, IQ = Intelligence Quotient, ODI = Oxygen Desaturation Index, OSA = Obstructive Sleep Apnea, PSG = Polysomnography, PSQ-SRBD = Pediatric Sleep Questionnaire Sleep Related Breathing Disorder, PedsQL = Pediatric Quality of Life, PLMI = Periodic Limb Movement Index, REM = Rapid Eye Movement, SWA = Slow Wave Activity, SI = Snore Index, VAS QoL = Visual Analogue Scale Quality of Life

### Statistical Analysis

For the change score pair-wise meta-analysis, the Review Manager (RevMan) Software (Version 5.3) [25] was utilized, analyzing changes from baseline during the follow-up. The outcomes were continuous in nature and we executed a random-effects quantitative synthesis implementing the effect size of standardized mean difference (SMD) and calculated 95% confidence intervals (CIs) [26]. In the present review, a p-value of less than 0.05 indicated statistical significance.

The statistical heterogeneity was quantified using the I-squared measure and a p-value of less than 0.05 indicated statistical significance [27]. Heterogeneity was assessed as per Cochran’s Q test. As such, a I^2^ value between 0% and 40% indicated that heterogeneity might not have been important, a I^2^ value between 30 and 60% represented moderate heterogeneity, an I^2^ value between 50 and 90% showed substantial heterogeneity, and a I^2^ value between 75 and 100% represented considerable heterogeneity [28]. Of note, the presence of small study effects could not be assessed by means of funnel plots due to the limited number of included studies [29].

Aiming to further evaluate the effect of ATE and watchful waiting on OSAS in children depending on the severity of the initial disease, we divided the study population based on the mean AHI baseline measurements in mild OSAS, mild to moderate OSAS, and moderate or severe OSAS, and we conducted a subgroup meta-analysis, evaluating uniquely the change-score of AHI in those patients.

### Clinical Interpretation of the Results

For continuous data, Cohen’s rule of thumb was followed for the interpretation of effect sizes and the classification was as follows [30]:

− SMD < 0.4: small effect;
− 0.4 ≤ SMD < 0.7: moderate effect;
− 0.7 ≤ SMD: large effect.

## Results

### Literature Search

**Fig. 1:**
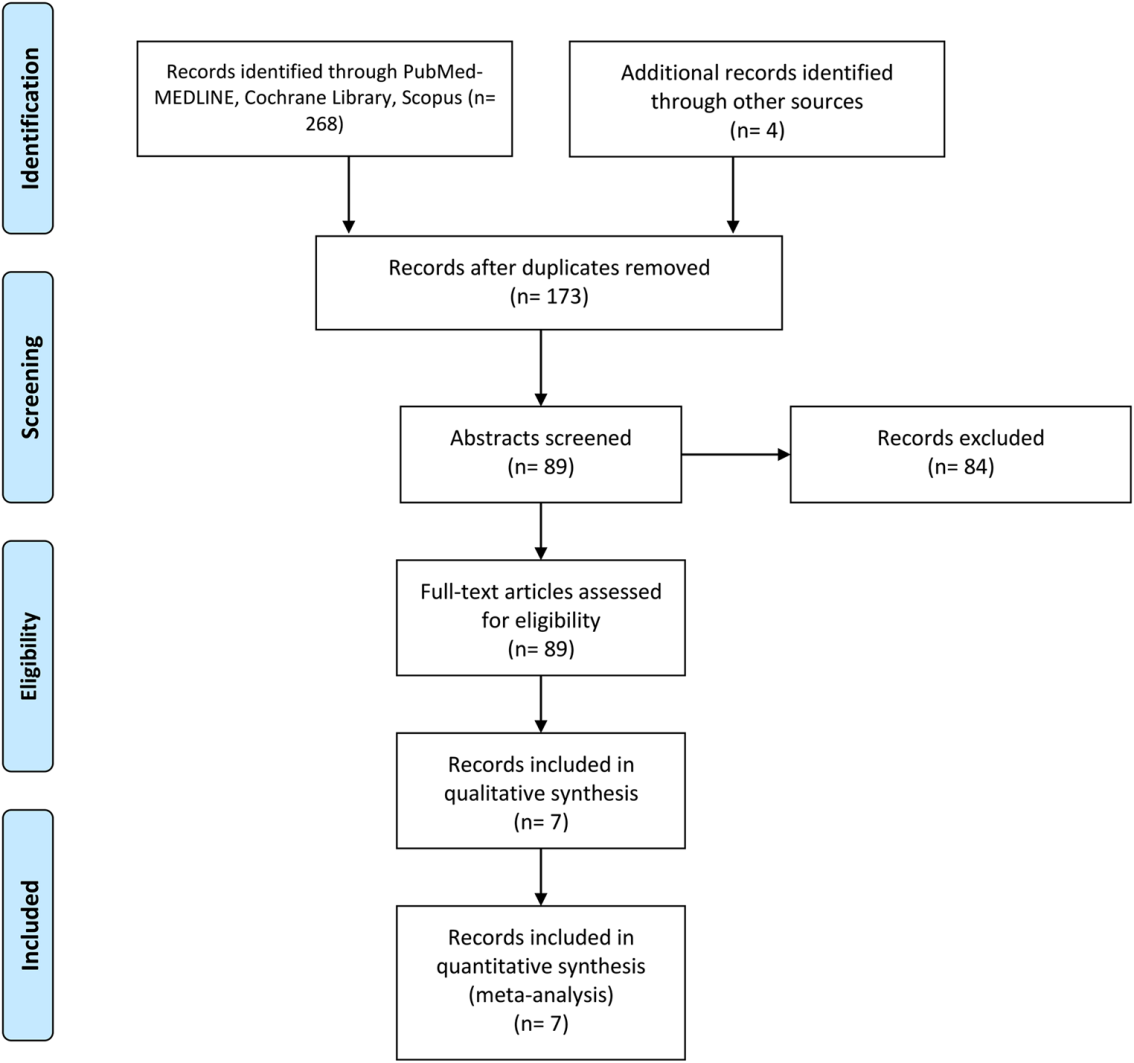
PRISMA flow diagram

268 articles were identified during the literature search and underwent deduplication using the Ovid deduplication tool (Fig. 1). After deduplication, the remaining 173 articles underwent title review by two independent reviewers (A.T. and S.A.), of which only 89 needed abstract review due to lack of relevance to our research questions to our study. Having read the abstracts, 21 studies were thoroughly analyzed and 7 were found eligible for inclusion in the meta-analysis.

### Study Characteristics

Five non-randomized and two randomized trials qualified for systematic reviewing. The enrolled studies were published between 2010 and 2020, with a total of 884 patients being eligible for qualitative synthesis. Two trials were conducted in US [31, 32], one in Spain [33], one in Sweden [34], one in Canada [35], one in Israel [36] and one in Taiwan [37]. Of note, the age of participants ranged between 1 and 16 years. Three papers reported on additional findings of the Marcus et al. trial [38–40] and were included in this review as part of this study. The follow-up period, namely the amount of time between the first and the second PSG, varied from 6 to 39 months, depending on the protocol of each study. Three studies assessed children with mild OSAS [31, 32, 37] two studies with mild to moderate OSAS [33, 34] and two studies with moderate or severe OSAS [35, 36] (Tables 1 and 2).

### Synthesis of the Results

#### Apnea-Hypopnea Index (AHI)

**Fig. 2:**
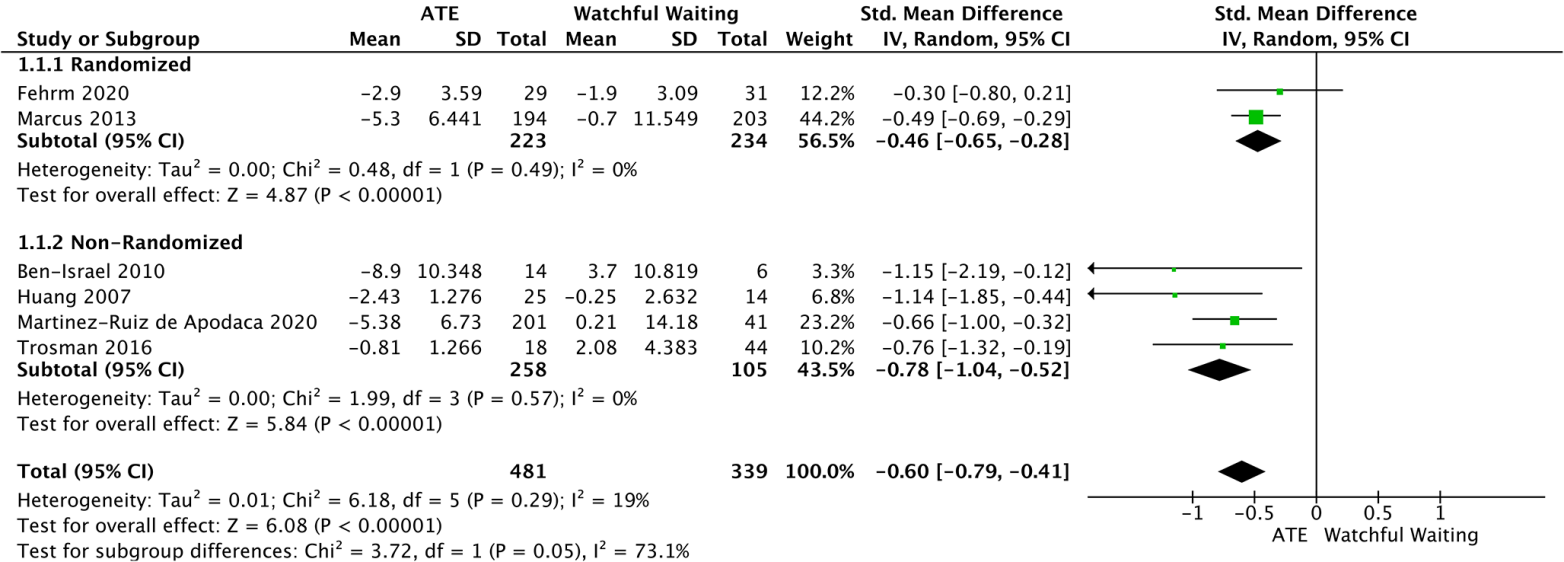
Forest plot for the assessment of AHI. Two different subgroups are considered. Vertical line demonstrates no difference between the two comparison groups. SMD = standardized mean difference, IV = inverse variance, SD = standard deviation, CI = confidence interval

AHI was assessed by data extracted from 2 randomized [34, 35] and 4 non-randomized trials [31, 33, 36, 37]. There was a statistically significant difference in favor of the ATE group compared to the watchful waiting group (SMD = −0.60, 95%CI −0.79 to −0.41, *p* < 0.0001). Of note, no important heterogeneity was detected (I2 = 19%; *p* = 0.29) (Fig. 2).

#### OSA-18

**Fig. 3:**
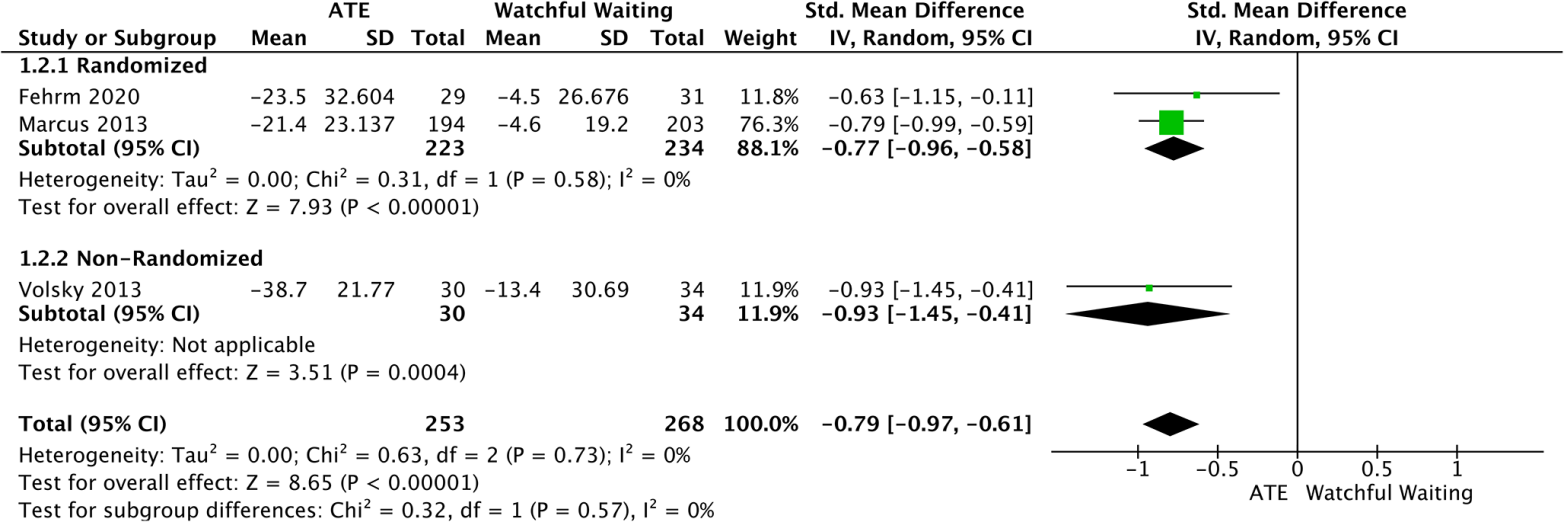
Forest plot for the assessment of OSA-18 score. Two different subgroups are considered. Vertical line demonstrates no difference between the two comparison groups. SMD = standardized mean difference, IV = inverse variance, SD = standard deviation, CI = confidence interval

OSA-18 score was assessed by data extracted from 2 randomized [34, 35] and 1 non-randomized trial [32]. ATE was statistically superior over watchful waiting (SMD = −0.79, 95%CI −0.97 to −0.61, *p* < 0.00001). No important heterogeneity was found (I2 = 0%; *p* = 0.73) (Fig. 3).

#### Mean SpO2

**Fig. 4:**
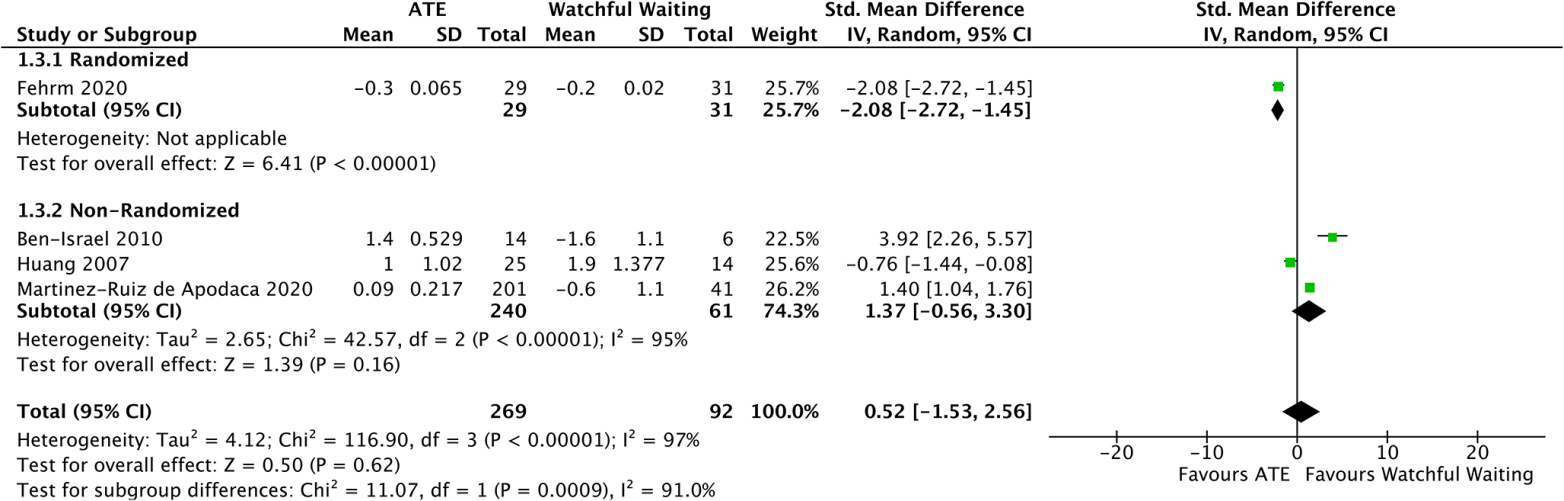
Forest plot for the assessment of SpO2 measurements. Two different subgroups are considered. Vertical line demonstrates no difference between the two comparison groups. SMD = standardized mean difference, IV = inverse variance, SD = standard deviation, CI = confidence interval

The changes in mean Sp02 measurements during polysomnography were assessed from 1 randomized [34] and 3 non-randomized trials [33, 36, 37]. The difference in favor of ATE was not found to be statistically important (SMD = 0.52, 95%CI −1.53 to 2.56, *p* < 0.62). It should be noted that substantial heterogeneity was detected (I2 = 97%, *p* < 0.00001) (Fig. 4).

#### Subgroup Analysis

**Fig. 5:**
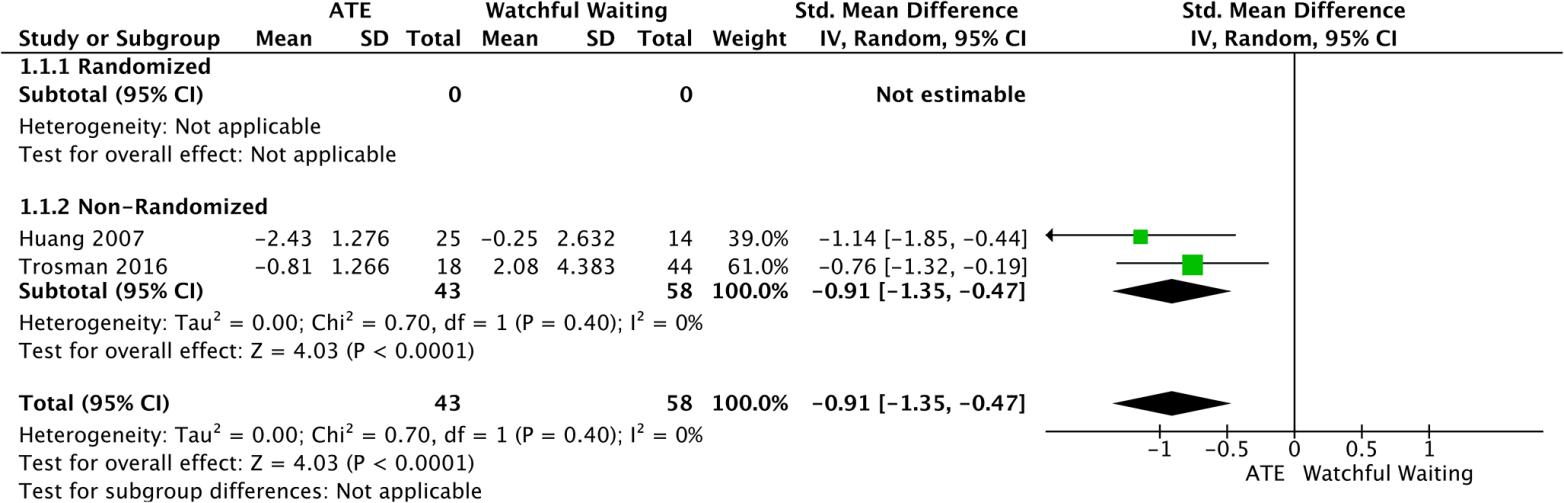
Forest plot for the assessment of AHI in children with mild OSAS. Two different subgroups are considered. Vertical line demonstrates no difference between the two comparison groups. SMD = standardized mean difference, IV = inverse variance, SD = standard deviation, CI = confidence interval

Mild pediatric OSAS was assessed based on AHI change-score by data extracted from 2 non-randomized trials [31, 37]. There was a significant difference in favor of ATE (SMD = −0.91, 95%CI −1.35 to −0.47, *p* < 0.0001). No important heterogeneity was spotted (I2 = 0%; *p* = 0.40) (Fig. 5).

**Fig. 6:**
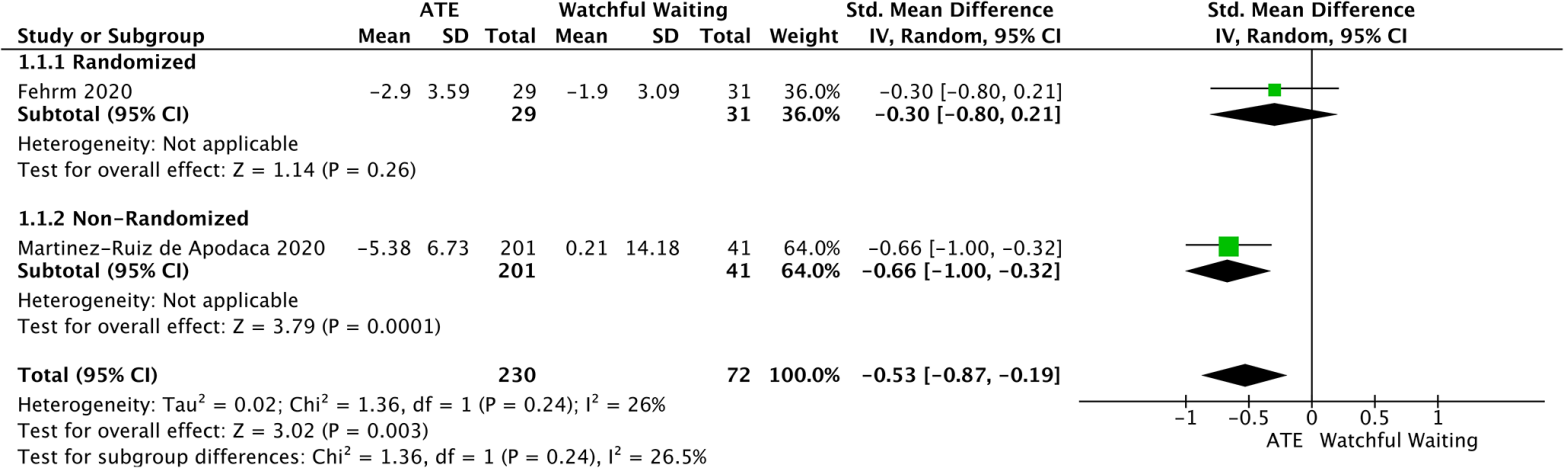
Forest plot for the assessment of AHI in children with mild to moderate OSAS. Two different subgroups are considered. Vertical line demonstrates no difference between the two comparison groups. SMD = standardized mean difference, IV = inverse variance, SD = standard deviation, CI = confidence interval

Mild to moderate pediatric OSAS was assessed based on AHI change-score by data extracted from 1 randomized and 1 non-randomized trial [33, 34]. There was also noted a significant difference in favor of AHI (SMD = −0.53, 95%CI −0.87 to −0.19, *p* < 0.003). No important heterogeneity was found (I2 = 26%; *p* = 0.24) (Fig. 6).

**Fig. 7:**
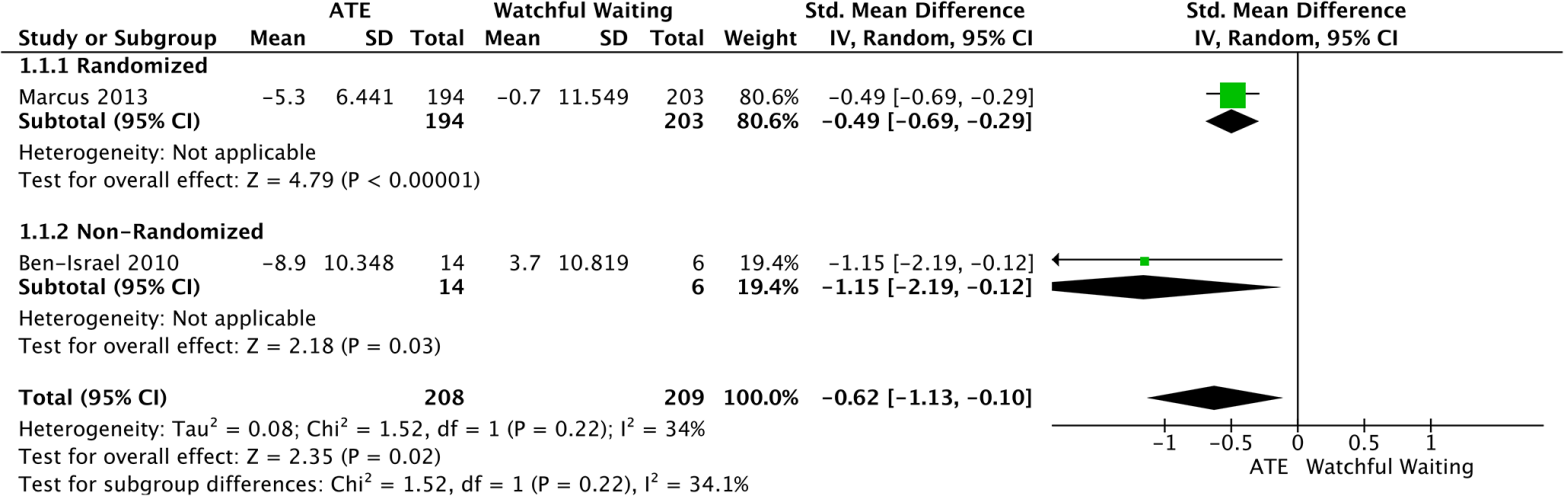
Forest plot for the assessment of AHI in children with moderate or severe OSAS. Two different subgroups are considered. Vertical line demonstrates no difference between the two comparison groups. SMD = standardized mean difference, IV = inverse variance, SD = standard deviation, CI = confidence interval

Moderate and severe pediatric OSAS was assessed based on AHI change-score by data extracted from 1 randomized and 1 non-randomized trial [35, 36]. No statistically significant decrease in change scores between ATE and watchful waiting was noted (SMD = −0.62, 95%CI −1.13 to −0.10, *p* = 0.02). Moderate heterogeneity was found (I2 = 34%; *p* = 0.22) (Fig. 7).

#### Quality Assessment

Overall, the two randomized trials were judged to be at low risk of bias [34, 35] (Table 3). All the included non-randomized trials were deemed to be at moderate risk of bias (Table 4) [31–33, 36, 37].

**Table 3:** Quality analysis of randomized trials utilizing the Cochrane risk of bias tool

Study	Randomization	Allocation concealment	Blinding of participants	Blinding of personnel	Blinding of outcome assessors	Incomplete outcome data	Selective outcome reporting	Other bias
Marcus (2013)	low	low	low	low	low	low	low	low
Fehrm (2020)	low	low	low	low	low	low	low	low

**Table 4:** Quality analysis of non-randomized trials utilizing the ROBINS-I tool

Study	Bias due to confounding	Bias in selection of participants into the study	Bias in classification of interventions	Bias due to deviations from intended interventions	Bias due to missng outcome data	Bias in measurement of outcomes	Bias in selection of the reported results	Overall Bias
Huang (2007)	Low	Low	Low	Low	Low	Moderate	Low	Moderate
Ben-Israel (2010)	Low	Low	Low	Low	Low	Moderate	Low	Moderate
Volsky (2013)	Low	Low	Low	Low	low	Moderate	Low	Moderate
Trosman (2016)	Low	Low	Low	Low	low	Moderate	Low	Moderate
Martinez-Ruiz de Apodaca (2020)	Low	Low	Low	Low	low	Moderate	Low	Moderate

## Discussion

The goal of the treatment of OSAS is to restore optimal breathing during the night to relieve associated symptoms, improve daytime functioning, and minimize negative impact. However, the applied interventions are varied, complex, and often multidisciplinary. Given the fact that hypertrophy of upper airway lymphadenoid tissues constitutes the most common factor underlying the presence of OSAS in children [11], guidelines from both the American Academy of Otolaryngology-Head and Neck Surgery (AAO-HNS) and the American Academy of Pediatrics (AAP) recommend adenotonsillectomy (ATE) as the first-line treatment [15].

However, ATE may only partially solve the problem of airway obstruction [15], since residual and recurrent disease is reported in a large proportion of children and can be attributed to multiple factors [41]. To elaborate further, persistent OSAS after ATE may occur between 13 and 29% among children categorized as low-risk patients, whereas in higher-risk groups such as children with craniofacial and upper respiratory malformations or extreme obesity, residual OSAS may be present in up to 75% [42]. Further risk factors for the persistence of OSAS after ATE include age > 7 years, asthma, allergic rhinitis, and the severity of OSAS prior to ATE [43].

Hence, taking into consideration the undeniable intraoperative and postoperative dangers of a surgical procedure with general endotracheal anesthesia in children, in addition to the usual phenomenon of residual OSAS after ATE, in this meta-analysis, we sought to compare ATE with the conservative watchful waiting strategy. Non-surgical treatment [11] such as weight loss, CPAP, intranasal steroids, and antileukotrienes could not be considered in this study because of the lack of relevant trials.

There is an increasing concern as to whether watchful waiting would be considered an appropriate if not recommended, alternative approach depending on the severity of the disease. Coincident with this perspective, data from the CHAT [35] study suggest that young children with non-severe OSAS and the absence of sleep-related complications could be managed non-surgically. Although this trial provided evidence for the beneficial effects of early ATE, including improvements in symptoms, parent-reported behavior, quality of life, and polysomnographic findings in the treatment group, polysomnographic abnormalities were also resolved in 46% of the children in the watchful waiting group. This means that half the children in the watchful-waiting group showed normalization of AHI [35]. Additionally, this conservative watchful waiting approach is affirmed by a study by Calhoun et al. [44] which demonstrated that children with mild OSAS have neurocognitive functioning equivalent to control patients. Furthermore, proponents of the watchful waiting claim a low prevalence of tonsillar hypertrophy after children turn 8 years old [44].

Contrary to the former findings, a network meta-analysis assessed the effectiveness of various interventions for pediatric OSAS. Fourteen comparative studies involving 1064 otherwise healthy children with adenotonsillar hypertrophy were included. The study concluded that ATE was still the most effective intervention compared with no treatment in terms of improvement in AHI [45]. Furthermore, Katidis et al. [46] concluded that ATE may be beneficial in children with 1–5 events/h associated with the following conditions: cardiovascular or central nervous system-associated morbidity, enuresis, somatic growth delay or growth failure, decreased quality of life, and persistent risk factors for OSDB.

For mild OSAS especially, limited evidence is available regarding the outcomes in children with mild OSAS who have not undergone ATE. A systematic review by Tan et al. showed that mild OSAS is relieved in approximately two-thirds of the children as they grow older [47]. In any case, children with tonsillar hypertrophy should be monitored closely for the early detection of worsening OSAS [48]. On the other hand, in a prospective cohort study, Li et al. reported that not only there was no spontaneous resolution of mild OSAS in untreated children, but also a worsening in 29% after two years of follow-up [48]. Moreover, another prospective trial demonstrated significant improvement in pediatric patients undergoing ATE for mild OSAS compared to the controls, regarding the quality of life, at a follow-up of 8 months [32]. An aggressive surgical approach is further encouraged by several studies that present neurocognitive deficits in children with only mild airway obstruction, including primary snoring [49, 50].

All in all, this study proved clinically the superiority of surgical intervention compared to a conservative watchful waiting strategy in terms of improvement of the AHI and OSA-18 score. Surprisingly enough, the improvement regarding the mean SpO2 score between the ATE and watchful waiting group was not found to be clinically higher in the former. However, this finding could be justified by the substantial heterogeneity that was detected.

What children with mild and mild to moderate OSAS concerns, for which the therapeutic strategy is still arguable, the subgroup analysis indicated significant clinical improvement of OSAS in children subjected to ATE, as indicated through the improvement of the AHI. On the contrary, it was unexpected that the clinical difference between moderate and severe OSAS in children subjected to surgical treatment was not significant, but again moderate heterogeneity was present in this comparison.

Thus, this systematic review and meta-analysis support the notion that ATE could be justified in most of the patients without contraindications, compared to watchful waiting.

## Limitations

We recognize that this study is not without limitations. First, the level of evidence we have provided was of moderate strength, due to the inclusion of non-randomized studies. Second, in the current meta-analysis we only considered three outcome measures, that is AHI index, OSA-18 score, and mean SpO2 levels, which reflects the fact that limited data were available for statistical pooling. To elaborate, investigating further parameters such as the Modified Epsworth Sleepiness Scale, Pediatric Sleep Questionnaire (PSQ), oxygen desaturation index (ODI), arousal counts, or Sleep-Related Breathing Disorder Scale (PSQ-SRBD) would have been of the essence for us to draw more robust conclusions on ATE clinical outcomes. Furthermore, the provision of neurocognitive measurements would have helped a great deal to determine the clinical relevance of the findings of this meta-analysis. Last but not least, synthesizing data from particular subgroups such as patients with Trisomy 21, craniofacial syndromes, obesity, and different ethnic backgrounds would have helped in delineating the extent of residual OSAS following ATE.

## Conclusion

The current meta-analysis provides evidence of moderate strength which supports surgical treatment over watchful waiting for the management of pediatric OSAS. Further RCTs are required to strengthen the results of this study.

## Electronic Supplementary Material

Below is the link to the electronic supplementary material.

Supplementary Material 1
