# Supplementary material for: Adenotonsillectomy Versus Watchful Waiting for Children with Obstructive Sleep Apnea Syndrome: A Systematic Review with Meta-Analysis
Source: Indian J Otolaryngol Head Neck Surg. 2024 May 20;76(5):4910–22. doi: 10.1007/s12070-024-04738-0 (PMC11456012; doi:10.1007/s12070-024-04738-0)
Supplement: Supplementary file 1 — Supplementary Material 1 [file 12070_2024_4738_MOESM1_ESM.docx]

The keywords used for the full search strategy are “adenotonsillectomy”, “tonsillectomy”, “obstructive sleep apnea”, “sleep apnea”, “watch and wait”, “watchful waiting”, and “observation”.
